# Identification of metabolomics-based biomarker discovery in individuals with down syndrome utilizing kernel-tree model-enhanced explainable artificial intelligence methodology

**DOI:** 10.3389/fmolb.2025.1567199

**Published:** 2025-04-09

**Authors:** Cemil Colak, Fatma Hilal Yagin, Burak Yagin, Abedalrhman Alkhateeb, Mahmood Basil A. Al-Rawi, Moulay A. Akhloufi, Mohammadreza Aghaei

**Affiliations:** ^1^ Department of Biostatistics and Medical Informatics, Faculty of Medicine, Inonu University, Malatya, Türkiye; ^2^ Department of Computer Science, Lakehead University, Thunder Bay, ON, Canada; ^3^ Department of Optometry, College of Applied Medical Sciences, King Saud University, Riyadh, Saudi Arabia; ^4^ Perception, Robotics and Intelligent Machines (PRIME) Lab, Department Computer Science, Université de Moncton, Moncton, NB, Canada; ^5^ Department of Ocean Operations and Civil Engineering, Norwegian University of Science and Technology (NTNU), Alesund, Norway; ^6^ Department of Sustainable Systems Engineering (INATECH), Albert Ludwigs University of Freiburg, Freiburg, Germany

**Keywords:** down syndrome, metabolomics analysis, biomarker, machine learning, SHAP, KTBoost

## Abstract

Objective: This study aims to develop an explainable artificial intelligence (XAI) model integrated with machine learning (ML) to comprehensively investigate metabolic differences between individuals with Down syndrome (T21) and healthy controls (D21) and to identify novel/pathway-specific biomarkers. In this study, ML classifiers including AdaBoost, LightGBM, Random Forest, KTBoost, and XGBoost are applied to metabolomics data obtained from metabolomic analyses by high-resolution liquid chromatography-mass spectrometry (LC-MS) using blood plasma samples of 316 T21 and 103 D21 individuals, and the importance of metabolites is evaluated by XAI-based SHAP analysis. The KTBoost model shows the highest classification performance with an accuracy of 90.4% and area under the curve (AUC) of 95.9%, outperforming AdaBoost, LightGBM, Random Forest, and XGBoost. Significant downregulation and upregulation of some metabolites were observed in the T21 group compared to the D21 group. Metabolites such as vitamin C, taurolithocholic acid, sphingosine, and prostaglandin A2/B2/J2 are observed at low levels in the T21 group. In contrast, metabolites such as thymidine, tau-roursodeoxycholic acid, serine, and nervonic acid are elevated. SHAP analysis revealed that L-Citrulline, Kynurenin, Prostaglandin A2/B2/J2, Urate, and Pantothenate metabolites could be novel/pathway-specific biomarkers to differentiate the T21 group. This study revealed significant metabolic alterations in individuals with T21 and demonstrated the effectiveness of the combination of ML and XAI methods to identify novel/pathway-specific biomarkers. The findings may contribute to a better understanding of Down syndrome’s molecular mechanisms and the development of future diagnostic and therapeutic strategies.

## Graphical Abstract

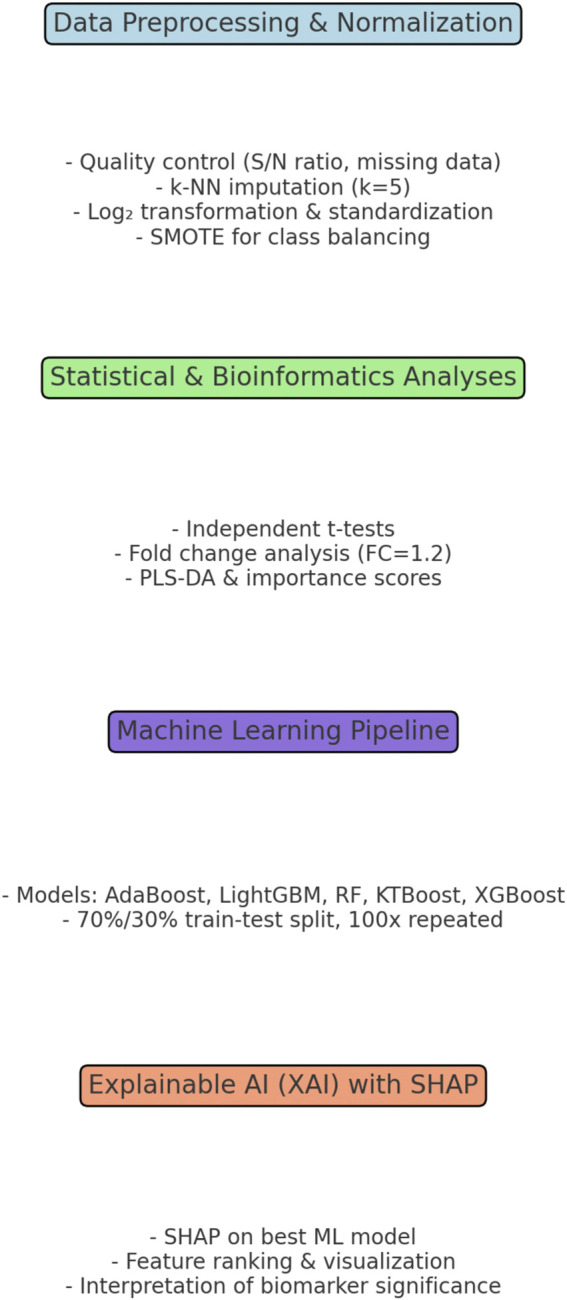

## 1 Introduction

Down syndrome (DS) is a genetic disorder due to trisomy of chromosome 21 and is associated with intellectual disability, characteristic facial features, and secondary conditions. DS is the most common chromosomal abnormality, with an incidence of approximately one in every 700 live births worldwide. Multiple physiological and metabolic changes are characteristics of the syndrome and may have striking effects on the quality of life of affected individuals. Metabolomic studies have played an important role in DS in recent years in understanding the molecular mechanisms in DS and discovering some biomarkers. Metabolomics is a powerful tool for comprehensively analyzing small biological molecules (metabolites) and understanding the disease process better. Metabolic alterations seen in DS may offer new insights into the syndrome’s pathophysiology and lead to early diagnosis, prognosis, and treatment strategies (Bahado-Singh et al., 2015; Pecze et al., 2020).

In recent years, metabolomic studies have become increasingly important to understand the molecular mechanisms underlying DS and to identify novel/pathway-specific biomarkers. Metabolomics is a powerful tool for comprehensively analyzing small molecules (metabolites) in biological systems, providing a better understanding of disease processes. Metabolic alterations observed in DS may shed light on the syndrome’s pathophysiology and contribute to developing early diagnosis, prognosis and treatment strategies. Abnormalities in various metabolic pathways have been observed in individuals with DS, and defects in mechanisms such as oxidative stress and antioxidant defense have been widely reported. Altered levels of vitamin C and other antioxidants have been associated with increased oxidative damage, which is frequently observed in individuals with DS.

Furthermore, abnormalities in lipid metabolism, particularly sphingolipid and cholesterol metabolism, have been identified. These changes are associated with the neurological symptoms observed with DS and the risk of early-onset alzheimer’s disease. Alterations in amino acid metabolism also play an important role in DS. For example, disturbances in homocysteine-related metabolic pathways have been associated with an increased risk of cardiovascular disease. Furthermore, alterations in tryptophan metabolism and the kynurenine pathway may contribute to the immunological and neurological abnormalities observed in DS. Defects in energy metabolism and mitochondrial function are also important features of DS. Changes in the levels of metabolites such as pantothenic acid (Vitamin B5) may indicate mitochondrial dysfunction and contribute to the various clinical features observed in DS. Inflammatory processes and immune system dysregulation are also important features of DS. Alterations in the levels of inflammatory mediators such as prostaglandins may be associated with chronic inflammation and susceptibility to autoimmune diseases observed in individuals with DS (Pecze and Szabo, 2021; Dierssen et al., 2020; Kiluk et al., 2021). However, the pathogenesis of DS is complex, and the multiplicity of contributing factors, overfitting and instability make it difficult to identify important biomarkers using only traditional statistical methods.

Metabolomics research has attempted to determine markers for DS through evaluations of oxidative stress together with lipid metabolic pathways and mitochondrial dysfunctions. First-trimester DS predictions through metabolomics profiling became possible after a medical paper showed that changes in amino acids and lipids indicated early signs of the syndrome (Bahado-Singh et al., 2013). A clinical article established that Down syndrome produced widespread disturbances in bioenergetic pathways along with impairments in tricarboxylic acid (TCA) cycle intermediate function and impaired mitochondrial activity, which leads to DS neurodevelopmental and cardiac impairments (Pecze and Szabo, 2021). Multiple studies confirm that DS is associated with decreases in vitamin C and glutathione antioxidants because of genetic overexpression of the SOD1 gene located on chromosome 21. Studies revealed insulin signaling along with problems in lipid metabolism as crucial elements in DS pathway development, which suggests therapists could utilize ceramides and phospholipids as diagnostic markers (Muchová et al., 2014). The existing research faced two main limitations because it used univariable statistical examination on small sample groups, which prevented them from studying intricate metabolic network relationships. Research presented machine learning (ML) to effectively combine Alzheimer’s-related DS biomarkers through multi-omics data integration. The lack of interpretation clarity currently stands in the way of doctors adopting this approach into clinical practice. The research fills current scientific voids through its integration of extensive metabolomics analysis with explainable artificial intelligence XAI solutions, which lead to both new biomarker discovery and practical discoveries about DS disease origins (Dierssen et al., 2020; Petersen and O’Bryant, 2019).

In recent years, ML algorithms have been increasingly used in the detection of complex diseases and analysis of omics data such as metabolomics. These approaches offer powerful tools for analyzing complex metabolic profiles and identifying novel/pathway-specific biomarkers. In the literature, algorithms such as KTBoost (a hybrid kernel-tree boosting algorithm), XGBoost (gradient-boosting algorithm), and Random Forest have been reported to perform highly in discriminating diseases using omics panel data (Yagin et al., 2024a; Yagin et al., 2023a; Yagin et al., 2023b). ML prediction models provide significant advances in diagnosing genetic diseases and biomarker discovery. Çelik et al. (2017) identified Down syndrome genes with high accuracy by analyzing protein levels. Complementing this work, Petersen and O'Bryant (2019) examined blood biomarkers for detecting Alzheimer’s disease in individuals with Down syndrome and obtained promising results. Asif et al. (2018) were successful in identifying genes in complex diseases such as Autism Spectrum Disorder using Gene Ontology. This approach seems to be applicable to other genetic disorders. In the field of image processing, Pooch et al. (2020) achieved high accuracy rates in the automatic detection of Down syndrome using facial features.

Zhang et al. (2021) made significant advances in biomarker discovery using gene expression data. The PermFIT method developed in one study improved the prediction accuracy of ML classifiers by identifying important biomarkers in complex human diseases such as Down syndrome. In conclusion, machine learning techniques have shown promising results in the fields of genetic disease detection, biomarker discovery and disease prediction and are expected to become more important in the future (Khalsan et al., 2022).

However, with the loss of confidence in standard machine learning classifiers due to their lack of interpretability (Krishnan, 2020), emerging explainable artificial intelligence (XAI) excels at processing high-dimensional data such as metabolomics and provides better generalization and differentiation capabilities, especially in the assessment of patient health and complications (Bansal et al., 2021; Ribeiro et al., 2016). The use of XAI is designed to make it easier to understand and diagnose the model output, no matter how accurate the output is. As a result, it will help users understand the results of the system and provide the model developer with insightful input to improve the model (Utomo et al., 2023; Pratap et al., 2023).

Despite the success of standard classifiers in several DS investigations, more research needs to be done on the application of XAI in DS. Therefore, XAI-based research can enhance our understanding of the complex pathogenesis of DS and aid in the development of diagnostic and treatment strategies. XAI-based models have the potential to reveal previously unknown biomarkers, as well as improve diagnostic sensitivity, which leads to more effective and personalized treatment (Cansel et al., 2023). XAI methods such as SHapley Additive exPlanations (SHAP) are essential for translating metabolomics data into clinically actionable information. XAI approaches address the “black box” problem, which increases trust in ML models, enables rapid validation of biomarkers, and speaks to the growing interest in transparent AI approaches in biomedical research. The use of XAI in this study is a concrete example of the paradigm shift toward interpretable, mechanism-driven approaches in DS research.

The present study seeks to investigate the metabolic differences between individuals with DS and controls using high-resolution metabolomics profiling and analytics to identify novel metabolomics biomarkers. The aim of the proposed framework is to explain the molecular mechanisms of DS at the pathophysiological level using integrated bioinformatics-based methodologies and tree-based, machine learning classifiers, including AdaBoost, LightGBM, XGBoost, KTBoost, and Random Forest, complemented by XAI through SHAP analysis. This integrated approach aims to standardize the assessment of classifier performance, improve model explainability and provide a solid prediction framework for DS.

## 2 Materials and methods

The methodology of this study is based on the STROBE guideline and is described below in accordance with the guideline.

### 2.1 Study design, participants and variables

The open-access data used in this study are available on the NIH Joint Fund’s National Metabolomics Data Repository (NMDR) website, Metabolomics Workbench (www.metabolomicsworkbench.org), where the project ID is designated as ST002200. Detailed information about the study design and data collection methods can be found in the Metabolomics Workbench entry for the project and in prior publications from the Human Trisome Project. The data can be accessed directly via it's Project DOI: (10.21228/M8C99T), the original project was supported by NIH grant, U2C- DK119886. The Inonu University Health Sciences Non-Interventional Clinical Research Ethics Committee approved this study (approval number: 2024/6496). The research from which the dataset was taken was conducted in a cross-sectional design to compare the metabolic profiles of individuals with DS (T21 group) and healthy individuals (D21 group). In the related study, metabolomics data from the T21 and D21 groups were collected at a single time point. The current study focuses on analyzing the relative abundance of available metabolites in the blood plasma of 316 individuals with T21 and 103 healthy controls (419 in total) (Powers et al., 2019).

Participants were carefully selected to ensure the validity of the study. The T21 group consisted of individuals diagnosed with Down syndrome and individuals in the D21 group were selected from healthy individuals without any known neurological or metabolic diseases. All participants were matched for demographic characteristics including gender and age, thus reducing the influence of potential confounding factors. These selection criteria ensure that the metabolic profiles of individuals in both groups reflect only Down syndrome-specific differences (Zhang et al., 2021).

Down syndrome status (T21 or D21) and specific metabolite levels obtained by metabolomics profiling were considered as primary outcome variables. Metabolite levels were evaluated as possible biomarkers affecting the T21 group in particular.

### 2.2 Power analysis

A total of 419 cases, including 316 T21 individuals and 103 healthy controls, were evaluated in this study. The sample size required for this study was determined using MetSizeR (https://cran.r-project.org/web/packages/MetSizeR/index.html accessed on 1 March 2024) using the probabilistic principal component analysis (PPCA) model. The calculation was based on a false discovery rate of 0.05. As a result, a minimum sample size of 14 patients was determined to be required, with 7 patients in each group. Although it was challenging to recruit T21 patients and healthy controls who met the specific inclusion criteria outlined in this research, the sample size exceeded the estimate obtained using MetSizeR, a method commonly used to assess sample size in metabolomics studies.

### 2.3 Data analysis, modeling and performance evaluation

#### 2.3.1 Data preprocessing and normalization

The raw data obtained from metabolomics analyses were first subjected to a quality control process. In this process, metabolites with a signal-to-noise ratio below three (Pecze and Szabo, 2021) and samples with more than 30% missing data were excluded from the analysis. The remaining missing data were filled using the *k*-nearest neighbor (*k*-NN) algorithm (*k* = 5). The conformity of the data to normal distribution was assessed using the Shapiro-Wilk test. Non-normally distributed data were log_2_ transformed to stabilize variance and attenuate skewness, an approach commonly applied in metabolomics to address heteroscedasticity. Finally, all data were standardized with the auto-scaling method. Synthetic Minority Oversampling Technique (SMOTE) approach was applied to address the problem of class imbalance between groups for the output variable. To limit the risk of excluding relevant features, the study focused on preserving the entire metabolite profile for ML analysis.

#### 2.3.2 Statistical and bioinformatics analyses

Independent sample t-tests were performed to determine the differences in metabolite levels between T21 and D21 groups. Metabolite data were normalized by log-transformation during the analysis process, thus homogenizing the distribution of the data. In analyzing metabolite levels, fold change analysis was applied to compare groups and a volcano plot was drawn. A threshold value of fold change (FC) = 1.2 was used to identify metabolites showing significant differences; this value is widely preferred in the literature for the detection of metabolites showing statistically significant up- and downregulation. The level of statistical significance was set at *p* < 0.05. A partial least squares-discriminant analysis (PLS-DA) model was used to assess overall differences in metabolite profiles. The PLS-DA model was performed based on 10-fold cross-validation, and important metabolites were visualized using variable importance scores. All p-values were adjusted using the Benjamini–Hochberg procedure (DeLong et al., 1988). DeLong’s test was utilized for the comparison of the areas under correlated receiver operating characteristic curves.

#### 2.3.3 Machine learning algorithms

Five different ML algorithms, namely, AdaBoost, LightGBM, RF, KTBoost and XGBoost, were used to compare the performance of classifying T21 and D21. AdaBoost, LightGBM, RF, KTBoost, and XGBoost methods are algorithms to improve classification performance using ensemble learning and various ML strategies. AdaBoost builds a strong model by sequentially training weak classifiers (usually decision trees) and giving more weight to errors at each step (Freund and Schapire, 1997). LightGBM is a gradient-boosting algorithm that works fast and efficiently on large datasets; it uses histogram-based approaches to data sampling. RF is an ensemble model that combines multiple decision trees and classifies based on the vote of each tree, reducing the risk of overlearning (Ahn et al., 2023; Guldogan et al., 2023). KTBoost combines boosting and kernel methods, leveraging both strengths to capture complex, non-linear relationships within the data (Sigrist, 2021). XGBoost uses optimization and parallelization techniques to speed up the gradient boosting process and improve accuracy, typically offering low and high memory usage (Yagin et al., 2024a; Yagin et al., 2024b). These methods use different optimization and weighting strategies to improve classification performance, resulting in robust and reliable models. All models were implemented using Python 3.9 and the Scikit-learn 1.4.2 library. The dataset was divided into 70% training set and 30% test set, and then this process was repeated 100 times, the performance of the models is expressed as the average of these 100 repetitions. Calculating accuracy, sensitivity, specificity, F1 score, AUC, and Brier score metrics evaluated the performance of the models.

#### 2.3.4 Explainable artificial intelligence

XAI is the general name for methods developed to make artificial intelligence and machine learning models more transparent and understandable. Although traditional machine learning models, especially deep learning-based models, offer high accuracy and performance, their decision-making processes often remain a “black box” due to their complexity. XAI improves the understandability of these “black box” models, enabling an understanding of why and how model outputs arise. These explanations allow users to assess the reliability of the model, transparently review decision-making processes and, if necessary, fine-tune the model to improve its performance. XAI is especially important for ethics, security and accuracy in decision-critical fields such as medicine, law, and finance. XAI methods make it possible to visualize the model’s decision processes, analyze the effects of certain features on the results, and ensure a balance of explainability and accuracy (Samek and Müller, 2019; Arrieta et al., 2020; Zhang et al., 2022).

In this study, SHAP analysis was applied in the XAI framework to distinguish the T21 group from healthy individuals and to identify metabolites prioritized as biomarkers by classification models. SHAP analysis was performed on the highest-performing ML model using Python’s SHAP library (version 0.39.0). SHAP values were calculated using the TreeExplainer method to visualize the decision-making processes of the model and to examine the effects of metabolites on classification. As a result of this analysis, metabolites were ranked according to their average absolute SHAP values, and the top 20 metabolites that stand out as the most effective biomarker candidates in distinguishing the T21 group were visualized in detail.

## 3 Results

According to FC analysis results, Vitamin C, taurolithocholic acid, stearidonic acid, sphingosine, prostaglandin A2/B2/J2, pantothenic acid, eicosatetraenoic acid_1, docosahexaenoic acid, dihomog-linolenic acid/eicosatrienoic acid, cholic acid and some carnitine derivatives (CAR DC4: 0, CAR 8:1, CAR 6:0, CAR 5:1, CAR 5:0, CAR 5:0, CAR 5:0; OH, CAR 18:2, CAR 18:1, CAR 16:1, CAR 14:1, CAR 12:1, CAR 12:0, CAR 10:1, CAR 10:0). Therefore, these metabolites are at lower levels in the T21 group than in the D21. Taurolithocholic acid (1.49 fold decrease), sphingosine (1.64 fold decrease), pantothenic acid (1.74 fold decrease), EPA (1.46 fold decrease), prostaglandin A2/B2/J2 (4.36 fold decrease), cholic acid (1. 49-fold decrease), CAR 10:0 (1.44-fold decrease) and CAR 10:1 (1.33-fold decrease) were the metabolites with the highest fold change among the metabolites downregulated in the T21.

On the other hand, upregulation was observed for metabolites such as thymidine, tauroursodeoxycholic acid, serine, nervonic acid, heptylic acid, hypoxanthine, glycine, arginine, and some carnitine derivatives (CAR 16:1, CAR 14:1, CAR 12:0), indicating higher levels of these metabolites than in the D21. The upregulation in metabolites such as thymidine and tauroursodeoxycholic acid suggests that these components may play an important role in biological processes. Metabolites showing significant upregulation considering the FC 1.2 threshold include thymidine (12.282-fold), tauroursodeoxycholic acid (14.582-fold), serine (12.885-fold), nervonic acid (12.189-fold), heptylic acid (12.265-fold), hypoxanthine (14.202-fold), arginine (13.811-fold) and 2-aminobenzoic acid (16.627-fold) (Table 1).

**TABLE 1:** Fold change analysis results for biomarker candidate metabolites between T21 and D21 groups.

Metabolite name	FC	log_2_FC	p.Adjusted	log_10_p
Vitamin C	0.812	−0.299	0.025	15.989
Thymidine	12.282	0.296	<0.001	86.042
Tauroursodeoxycholic acid	14.582	0.544	<0.001	26.397
Taurolithocholic acid	0.669	−0.579	<0.001	15.157
Stearidonic acid	0.795	−0.329	0.001	29.309
Sphingosine	0.609	−0.714	<0.001	56.668
Serine	12.885	0.365	<0.001	77.363
Pyroglutamic acid	0.787	−0.344	<0.001	31.336
Prostaglandin A3/B3	0.824	−0.279	<0.001	26.888
Prostaglandin A2/B2/J2	0.229	−21.249	<0.001	76.325
Pantothenic acid	0.576	−0.796	<0.001	54.678
Octadecatrienoic acid	0.798	−0.323	0.036	14.407
N-Formyl-L-kynurenine	0.773	−0.371	0.014	18.461
Nervonic acid	12.189	0.285	<0.001	44.569
Lysophosphatidylinositol	0.788	−0.343	<0.001	87.034
Leukotriene B4/PGA1/PGB1	0.832	−0.265	<0.001	31.336
Kynurenine	0.762	−0.392	<0.001	81.201
Hypoxanthine	14.202	0.506	<0.001	5.817
Heptylic acid	12.265	0.294	<0.001	79.318
Glycine	12.187	0.285	<0.001	10.183
EPA	0.684	−0.546	<0.001	68.103
Eicosatetraenoic acid_1	0.757	−0.400	<0.001	54.478
Docosahexaenoic acid	0.762	−0.390	<0.001	32.563
Dihomo-g-Linolenic acid/eicosatrienoic acid	0.770	−0.376	<0.001	34.086
Deoxycholic acid	0.796	−0.328	<0.001	24.994
Cholic acid	0.670	−0.575	0.015	18.157
CAR DC4:0	0.802	−0.317	<0.001	54.678
CAR 8:1	0.778	−0.361	0.013	18.663
CAR 6:0	0.765	−0.385	<0.001	23.773
CAR 5:1	0.711	−0.491	0.013	18.663
CAR 5:0; OH	0.762	−0.391	<0.001	47.235
CAR 5:0	0.785	−0.348	<0.001	41.912
CAR 3:0	0.808	−0.306	<0.001	35.836
CAR 18:2	0.710	−0.493	<0.001	52.178
CAR 18:1	1.232	0.301	<0.001	47.235
CAR 16:1	12.128	0.278	<0.001	23.773
CAR 14:1	12.838	0.360	<0.001	23.011
CAR 12:1	13.567	0.440	<0.001	36.909
CAR 12:0	12.443	0.315	<0.001	22.612
CAR 10:1	0.752	−0.409	0.019	17.156
CAR 10:0	0.696	−0.521	<0.001	26.397
Arginine	13.811	0.465	<0.001	60.549
5-Hydroxyindoleacetic acid	0.755	−0.403	<0.001	86.731
2-Aminobenzoic acid	16.627	0.733	0.031	15.045
10(S)17(S)-DiHDHA/protectin D1	0.799	−0.323	<0.001	5.817

CAR DC4:0: Succinyl carnitine; CAR, 8:1: octenoyl-L-carnitine; CAR, 6:0: hexanoyl-L-carnitine; CAR, 5:1: Tiglylcarnitine; CAR, 5:0; OH: hydroxyvaleroyl carnitine; CAR, 5:0: valeroyl carnitine; CAR, 3:0: propionyl-carnitine; CAR, 18:2: Linoleyl carnitine/Linoelaidyl carnitine; CAR, 18:1: O-octadecenoyl-L-carnitine; CAR, 16:1: Hexadecenoyl-carnitine; CAR, 14:1:Tetradecenoyl carnitine; CAR, 12:1: O-dodecenoyl-carnitine; CAR, 12:0: L-Carnitine lauroyl ester; CAR, 10:1: O-Decenoyl-L-carnitine; CAR, 10:0: O-Decanoyl-L-carnitine.

**FIGURE 1:**
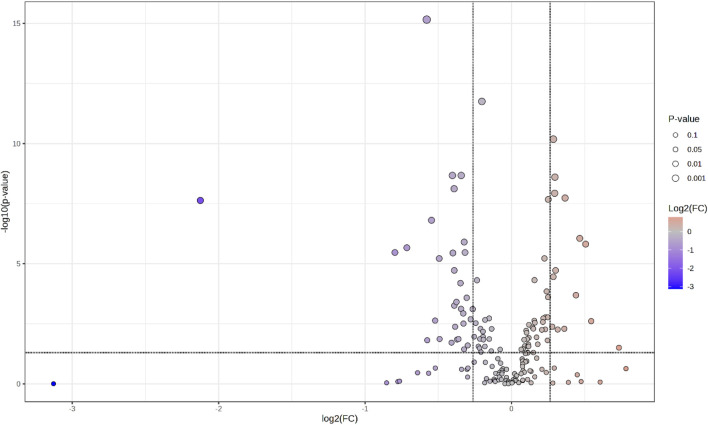
Volcano plot.

The volcano plot in Figure 1 provides an overview of the data along two important axes: log2(FC) (fold change value) and -log10 (p-value) (statistical significance). In the plot, the log2(FC) axis is horizontal and the log10 (p-value) axis is vertical. The vertical lines could represent log2(FC) = −0.263 and 0.263, reflecting the threshold FC = 1.2. Points to the left of these lines mean downregulation (FC < 0.833), while those to the right mean upregulation (FC > 1.2). The sizes of the dots represent the p-value, while the colors indicate log2(FC). The color gradient changes from darker shades of blue to shades of brown, indicating upregulation. Dots in shades of gray in the middle represent statistically insignificant changes, while the more distinctly colored dots, especially in the upper left and upper right, represent significant up- and downregulation. In this graph, the leftmost and rightmost points show the strongest regulation, while those with lower p-values are located more vertically, which implies stronger statistical significance.

**FIGURE 2:**
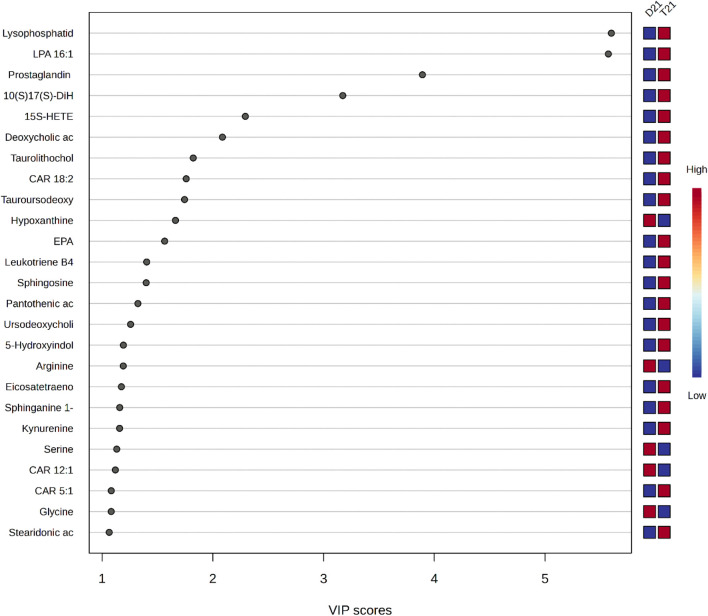
VIP graph for PLS-DA Model.

The PLS-DA model VIP plot (Figure 2) is based on VIP scores, which indicate the importance of metabolites in the model. Metabolites such as Lysophosphatidyl and LPA 16:1 have the highest VIP scores and contribute the most to the classification performance of the model. Other important metabolites include Prostaglandin, 10(S)17(S)-DiHDHA and 15S-HETE, which have a strong discriminative role in classification. Metabolites such as Stearidonic acid, Glycine and CAR 5:1 are relatively less effective in the model. The color scale reflects the levels of metabolites, with red shades indicating high levels and blue shades indicating low levels.

**FIGURE 3:**
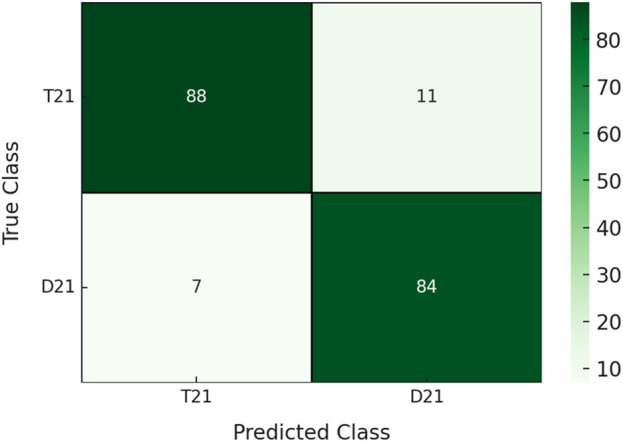
Confusion matrix of the KTBoost model for Down syndrome prediction.

According to the model performance evaluation results (Table 2), the highest accuracy and F1 score belong to the KTBoost model, with 90.4% and 93.1%, respectively. While KTBoost gives the best result in AUC values with 95.9%, XGBoost shows a close performance with 95.1%. In terms of sensitivity, XGBoost has the highest score at 96.6%, while the KTBoost model follows at 91.1%. In terms of specificity values, KTBoost offers a significantly higher result than the other models, with 88.8%. Finally, when the Brier scores are examined, it is observed that the KTBoost model achieved the lowest value at 5.9%, which shows that the model is more advantageous compared to the other models in terms of calibration accuracy. In general, the KTBoost model stands out as the most successful model in the classification task because it exhibits the best performance in accuracy, AUC, F1 score, specificity, and Brier score. The superiority of KTBoost based on AUC is statistically significant (DeLong’s test, p < 0.01 for XGBoost, LightGBM, and AdaBoost) after correcting for multiple comparisons using the Benjamini–Hochberg procedure. In contrast, Random Forest performed similarly (p = 0.682), likely as it is more stable owing to its ensemble approach (Table 2). Figure 3 presents the confusion matrix for the KTBoost model (Figure 3).

**TABLE 2:** Results of performance metrics for machine learning models.

Model	Accuracy	F1 score	AUC	Sensitivity	Specificity	Brier score
AdaBoost	0.896	0.929	0.920	0.955	0.750	0.195
LightGBM	0.880	0.918	0.941	0.933	0.750	0.100
RF	0.873	0.914	0.958	0.955	0.666	0.106
KTBoost	0.904	0.931	0.959	0.911	0.888	0.059
XGBoost	0.896	0.930	0.951	0.966	0.722	0.084

**FIGURE 4:**
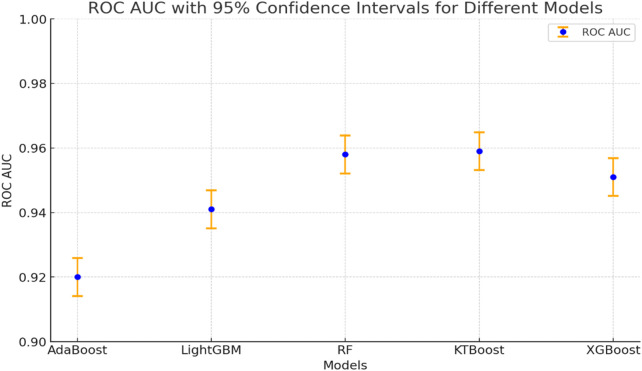
Different model results for ROC AUC values.

The performance evaluation of machine learning algorithms for differentiating T21 from D21 appears in Figure 4 through receiver operating characteristic (ROC) curves. Among the examined models, KTBoost demonstrated the best performance with a 95.9% AUC value; but XGBoost maintained an almost equivalent AUC value of 95.1%. LightGBM performed with an AUC at 94.1% and Random Forest achieved 95.8% while AdaBoost had a slightly lower AUC value of 92.0% among the compared models. The explanatory visualization demonstrates how tree-based ensemble methods especially KTBoost effectively recognize complicated metabolic patterns of DS with minor differences in model performance observed between top-ranking models (Figure 4).

**FIGURE 5:**
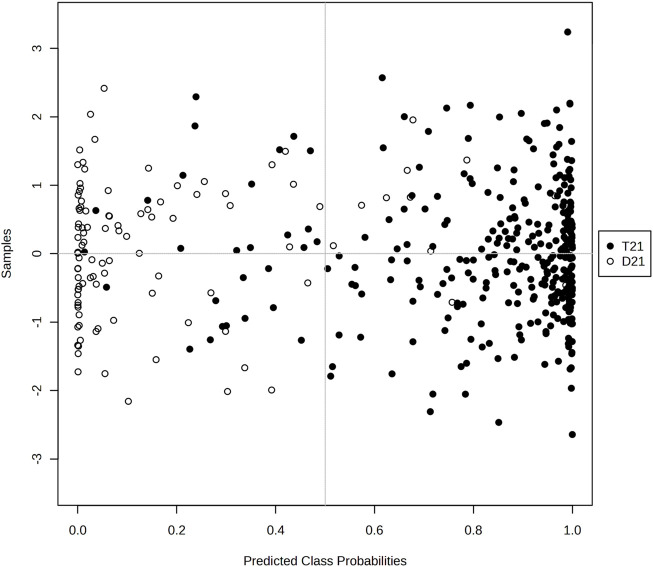
Graphical representation of the class probabilities of the optimal KTBoost model.

The graph of Figure 5 visualizes the distribution of the probabilities predicted by the model for the classes. The horizontal axis shows the class probabilities predicted by the model and the vertical axis shows the examples. Black filled circles represent the T21 class and white hollow circles represent the D21 class. In general, we can say that the model is successful in distinguishing the two classes and predicting the correct probabilities. Especially at the probability threshold of 0.5, the model is able to distinguish the two classes largely and the classification accuracy seems to be high (Figure 5).

**FIGURE 6:**
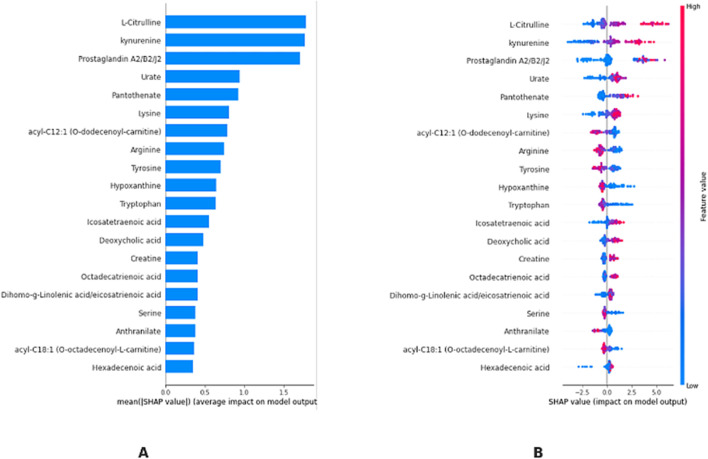
KTBoost model interpretation. **(A)**: Using the final model, we rank the stability and interpretative relevance of the top 20-biomarker metabolites **(B)**: Average order of importance (|SHAP value|) of the top 20 biomarker metabolites; the greater the SHAP value of a characteristic, the more probable the patient has T21.

Figure 6A shows the ranking of each metabolite in terms of mean SHAP value, highlighting their overall level of influence in the model. According to this ranking, the metabolites L-Citrulline, Kynurenin, Prostaglandin A2/B2/J2, Urate, and Pantothenate are included in the model as the most important possible biomarkers for differentiating T21. Figure 6B shows the effect of the KTBoost model on the classification decisions of the candidate biomarker metabolites through their SHAP values. This graph reflects the positive or negative contribution of each metabolite to the model outputs, assessing its importance through global SHAP values. Positive SHAP values indicate the contribution of the metabolite to the positive class (individuals with DS, T21), while negative SHAP values indicate the contribution to the negative class (healthy controls, D21). The dots in the image are colored with normalized values of the metabolites, with shades closer to blue representing low levels of metabolites and shades closer to pink representing high levels. L-Citrulline, Kynurenin, Prostaglandin A2/B2/J2, Urate, and Pantothenate play an important role in determining the positive class (T21) with high SHAP values. High levels of these metabolites increase the probability of T21 (Figure 6). Information summarizing the roles of biomarker candidate metabolites identified by XAI-assisted methodology and their relationship with DS and other genetic diseases is presented in Table 3 (Table 3).

**TABLE 3:** Key metabolites identified for down syndrome and their biological roles.

Metabolite	Biological role	Relevance to down syndrome and other genetic disorders
L-Citrulline	Involved in the urea cycle, nitric oxide metabolism, and endothelial function	Altered citrulline metabolism is linked to oxidative stress and endothelial dysfunction, which may contribute to vascular abnormalities in Down syndrome
Kynurenine	A key intermediate in tryptophan metabolism and modulator of immune response	Elevated kynurenine levels are associated with neuroinflammation and cognitive dysfunction, which are relevant to the neurological impairments seen in Down syndrome
Prostaglandin A2/B2/J2	Bioactive lipid mediators involved in inflammation and cellular signaling	Dysregulation of prostaglandins can contribute to immune dysregulation and increased inflammation observed in individuals with Down syndrome
Urate	An antioxidant that modulates oxidative stress and purine metabolism	Lower urate levels in Down syndrome may contribute to increased oxidative stress, a known factor in neurodegeneration and aging-related phenotypes
Pantothenate (Vitamin B5)	Essential for Coenzyme A synthesis, fatty acid metabolism, and energy production	Disruptions in pantothenate metabolism could impair mitochondrial function and energy metabolism, which are frequently altered in Down syndrome

## 4 Discussion

In this study, a comprehensive metabolomics analysis was performed using various machine learning classifiers integrated with XAI to examine metabolic differences between T21 and D21 groups and to identify novel/pathway-specific biomarkers. The results of the present study revealed significant metabolic differences between the T21 and D21 groups, indicating novel/pathway-specific biomarkers that could be used to characterize DS. FC analysis revealed significant up- and downregulation of some metabolites in the T21 group compared to the D21 group. In particular, vitamin C, taurolithocholic acid, sphingosine, prostaglandin A2/B2/J2, pantothenic acid, and various carnitine derivatives were downregulated. These findings suggest potential alterations in the metabolism or utilization of these metabolites in individuals with DS. These results are in line with metabolic changes observed in previously reported studies. The decrease in vitamin C may be associated with increased oxidative stress, which is often observed in individuals with DS. The decrease in taurolithocholic acid levels may indicate potential alterations in bile acid metabolism, which may be associated with gastrointestinal problems observed in individuals with DS (Muchová et al., 2014; Rueda and Martínez-Cué, 2020).

On the other hand, a marked upregulation of metabolites such as thymidine, tauroursodeoxycholic acid, serine, nervonic acid, hypoxanthine and arginine was observed. The increase in these metabolites may reflect alterations in cellular metabolism and signal transduction in DS. For example, the increase in thymidine may indicate potential alterations in DNA synthesis and repair, which may be associated with the genomic instability observed in DS. Increases in amino acids such as serine and arginine may indicate changes in protein metabolism. This may be associated with the neurological and immunological abnormalities observed in DS. Increased levels of nervonic acid may reflect potential alterations in myelin structure and function, which may be associated with the neurological symptoms often observed in DS (Coskun and Busciglio, 2012; Hetman and Barg, 2022a).

In this study, various ML classifiers (AdaBoost, LightGBM, Random Forest, KTBoost and XGBoost) were used to classify T21 and D21 groups. Among these models, KTBoost stood out with the highest accuracy (90.4%), F1 score (93.1%) and AUC value (95.9%). The high performance of the KTBoost model supports the potential use of metabolomics data in DS diagnosis. These results are in line with the performance of ML models developed by Hao et al. (2020) using metabolomics data. Comparing the performance of the models, KTBoost outperformed the other models, especially in terms of specificity (88.8%) and Brier score (5.9%). These results suggest that the KTBoost model has the ability to minimize false positives in DS diagnosis and better calibrate its predictions. KTBoost’s ability to maintain high accuracy while managing the complexities of high-dimensional data is further supported by its integration with advanced optimization techniques. The respective superior performance of KTBoost may be due to the combination of the tree-based structure of the model and the gradient boosting technique, which allows it to efficiently handle complex and high-dimensional metabolomics data (Khattak et al., 2023; Hussain et al., 2022).

SHAP analysis was used to improve the interpretability of model predictions and to identify the most effective metabolites. According to this analysis, the metabolites L-Citrulline, Kynurenin, Prostaglandin A2/B2/J2, Urate and Pantothenate stood out as the most important biomarker candidates to differentiate the T21 group. The high SHAP values of L-Citrulline suggest that this metabolite may play an important role in DS. L-Citrulline is an important intermediate in the nitric oxide (NO) cycle and is involved in vascular function and neurotransmission. Alterations in L-Citrulline levels in individuals with DS is likely associated with cardiovascular and neurological complications (Maric et al., 2021). This finding may help us understand the mechanisms underlying the increased cardiovascular risk and neurological abnormalities observed in individuals with DS. Alterations in kynurenine metabolism may contribute to the neurological and immunological abnormalities observed in DS. The kynurenine pathway is closely related to tryptophan metabolism and may affect neurotransmitter balance. Further investigation of the role of this metabolite in DS may provide new insights into the pathophysiology of the disease. Alterations in kynurenine metabolism may also be associated with neuropsychiatric symptoms often observed in DS, such as depression and cognitive impairments (Maric et al., 2021; Morita et al., 2013). The importance of prostaglandin A2/B2/J2 in model predictions points to the role of inflammatory processes in DS. Prostaglandins play a critical role in the regulation of inflammation and immune response. The chronic inflammation and susceptibility to autoimmune diseases observed in individuals with DS may be associated with altered levels of these metabolites (Hetman and Barg, 2022a; Yao and Narumiya, 2019). This finding may contribute to a better understanding of inflammatory processes in DS and the development of potential anti-inflammatory treatment strategies. Changes in urate levels may indicate potential abnormalities in oxidative stress and antioxidant defense mechanisms. Increased oxidative stress is commonly observed in DS and may be associated with complications such as neurodegeneration and premature aging. Given the antioxidant properties of urate, changes in the levels of this metabolite may be important in understanding the protective mechanisms against oxidative stress in DS. Alterations in pantothenate (Vitamin B5) metabolism may indicate potential abnormalities in energy metabolism and mitochondrial function. Mitochondrial dysfunction is commonly observed in DS and may contribute to various clinical features of the disease. Changes in pantothenate levels may help to better understand energy metabolism and mitochondrial function in DS and shed light on the development of potential therapeutic strategies (Nachvak et al., 2010).

XAI techniques, such as SHAP values, provide a powerful means for analyzing metabolite biomarkers for diagnosing T21. The current study selected SHAP over LIME because of its theoretical solidness through game-theory-based fair attribution methods along with its ability to work with tree-based models using TreeSHAP and its dual interpretability features for global biomarker rankings. SHAP provides quantitative measurements about metabolite effects such as elevated L-Citrulline levels increasing T21 risk; however, LIME does not offer this capability because its local approximation method suffers from inconsistent biological relevance. High SHAP values indicate a metabolite’s contribution to the model; but do not necessarily imply clinical relevance. As illustrated in the present study (Figure 5), the interpretation of the KTBoost model shows why some metabolites are more important than others in separating the cases of T21 from D21. Finally, the model ranks metabolites according to their mean SHAP values and L-Citrulline, Kynurenin, Prostaglandin A2/B2/J2, Urate, and Pantothenate are found to be the highest SHAP biomarkers. Not only does the SHAP value visualization quantify how important (or not) each metabolite is to the model, but also how they contribute to the model’s decision-making process (e.g., whether metabolite levels (high or low) lead to the model’s prediction of death or not). Through this approach, a particular metabolic profile of T21 can be understood more meaningfully, characterized by high concentrations of some metabolites increasing the probability of T21 diagnosis. Transparency in the AI-driven diagnostics is important for medical professionals to build trust—both for a clear rationale behind models predictions and for demand in the development of interpretable and accountable AI systems in healthcare (Zhang et al., 2023).

The clinical implications of these findings are pertinent to the progress of precision medicine in DS (T21). This case also highlights that identifying biomarkers like L-Citrulline or Kynurenine or Prostaglandin A2/B2/J2 could be used as a window for early diagnosis and as therapeutic targets, especially in the context of antioxidant treatment and chronic inflammatory management, which seem to be one of the many hallmarks of T21. Depletion of vitamin C and pantothenate was consistent with previous findings of mitochondrial dysfunction in T21, providing rationale for antioxidant supplementation as a potential ability (Rueda and Martínez-Cué, 2020; Baksh et al., 2023). The merging of XAI and metabolomics demonstrated the high accuracy (AUC = 95.9%) of the KTBoost model, which is a transparent framework that oncologists can trust and apply to AI diagnostic tools in practice. However, the application of these results to clinical practice is only possible if they are confirmed in longitudinal studies, taking into account the effects of confounding variables such as diet and comorbidities. Future studies should be concentrated on the interventional phases with the established pathways, including the kynurenine and nitric oxide cycles, for possible personalized therapies (Hetman and Barg, 2022b).Additionally, this study illustrates the power of ML-XAI workflows to discover metabolic adaptations with relevance to pathophysiology and to therapeutic avenues DS. The respective dysregulations of L-Citrulline (NO cycle), Kynurenine (tryptophan catabolism) and Prostaglandin A2/B2/J2 (inflammatory pathways) reflect a network of interconnected dysregulated metabolisms in DS. These pathways converge on oxidative stress and mitochondrial dysfunction, suggesting that therapeutic interventions targeting NO signaling or IDO inhibition might alleviate systemic comorbidities.

The results of the current research support previous investigations from the Human Trisome Project (Powers et al., 2019) by adding new understanding of DS metabolomics changes. The research studies recognize metabolic pathway and inflammatory pathway disorders; yet utilize different experimental designs. Through transcriptomic and proteomic profiling, the Human Trisome Project study determined that DS patients exhibited Alzheimer’s disease-related changes and immune dysregulation along with lipid metabolism disturbances. These findings established systemic inflammatory processes alongside neurodegeneration. The present study employed KTBoost machine learning together with SHAP analysis to locate the metabolite changes such as reduced vitamin C and prostaglandin levels in combination with elevated thymidine and nervonic acid levels. The current paper at metabolite resolution has added novel biomarkers including L-Citrulline, Kynurenine, and Urate to the biomarker set identified by the Human Trisome Project study. These research findings demonstrate how DS pathophysiology operates at detailed levels as the XAI method in this study enhances research benefits from the Human Trisome Project’s baseline multi-omics approach for discovering biomarkers and understanding biological processes (Powers et al., 2019).

The study results related to metabolic changes confirmed previously established T21 research findings. Research evidence supports the metabolic problems observed in this study since overexpressed SOD1 gene on chromosome 21 causes redox homeostasis disruption and antioxidant depletion in DS. The diminished presence of sphingosine together with carnitine derivatives (e.g., CAR 10:0) demonstrates earlier research on metabolic abnormalities and impaired mitochondrial function, which triggers neurodegenerative processes and energy deficits. Erroneous expression of thymidine enhances nucleotide levels for DNA repair since genomic instability occurs in T21 patients as a possible response to DNA damage. The research advances previous findings through its discovery of the biomarkers L-Citrulline and Prostaglandin A2/B2/J2, which connect, to nitric oxide pathways and chronic inflammation thus filling gaps in metabolomics study understanding. The research demonstrates that T21 causes widespread metabolic disturbances in the body and it offers new opportunities to treat multiple system-related consequences (Coskun and Busciglio, 2012; Yao and Narumiya, 2019). The associated metabolites are implicated in pathways involved in Down syndrome (DS) pathophysiology (L-Citrulline, Kynurenine, Prostaglandin A2/B2/J2, Urate, and Pantothenate). Abnormal regulation of their activity offers mechanistic pathways of the oxidative stress, mitochondrial dysfunctions, chronic inflammation and neurodegenerative processes, which are represented as the main characteristics of DS. Collectively, these biomarkers underscore several interconnected pathways—oxidative stress, inflammation, and mitochondrial dysfunction—that likely contribute to DS pathogenesis. Identifying those via ML-XAI brings actionable targets for diagnostic panels and therapeutic interventions, providing critical translational bridges between metabolomics and clinical application in DS research. This study validates known metabolic disturbances uncovered in DS, including vitamin C depletion (oxidative stress) and decreased pantothenic acid (mitochondrial dysfunction), corroborating data from the mentioned scientific studies earlier. We offer new meta-information, such as thymidine upregulation (a DNA repair compensation) and prostaglandin A2/B2/J2 downregulation, indicative of inflammatory dysregulation and expanding the metabolic signature of DS. These findings not only confirm the known pathways but also report new biomarkers, affirming the potential of plasma metabolomics for DS profiling in a noninvasive manner compared to previously mentioned investigations based on tissue/urine.

Although the findings of this study provide important contributions to better understanding the metabolomics characteristics of individuals with DS, their generalizability to large populations may be limited. Future studies should perform external validation studies using larger and diversified sample groups to increase the generalizability and validity of these findings for clinical applications. As our study was limited to a cross-sectional design, further longitudinal studies are recommended to support these findings. Such studies may help us understand the dynamics of metabolic changes in DS over time and their relationship with clinical manifestations. Furthermore, given the phenotypic heterogeneity of DS, studies on different phenotypic subgroups are needed. Such subgroup analyses may enable more precise identification of specific metabolic alterations and provide more in-depth insights into the phenotypic diversity of DS. In order to better control for the effects of environmental factors, especially diet and lifestyle, on the metabolomics profile, further evaluation of these variables is recommended. In addition, future studies could further explore the consistency of biomarker identification by comparing feature importance across different machine learning models, providing additional insights into the robustness and generalizability of the identified biomarkers. Finally, using more advanced and sensitive technologies in metabolomics analyses may increase the likelihood of detecting metabolites with low concentrations or those prone to degradation. Such advanced analysis methods could contribute to a more comprehensive and detailed understanding of the pathophysiological mechanisms underlying DS (Dierssen et al., 2020; Baksh et al., 2023; Hendrix et al., 2021). Although demographic information (sex, age) was matched between T21 and D21 groups at the time of participant selection, it was not directly incorporated as covariates in the ML models. This reflects a potential for residual confounding, since metabolic profiles may differ according to age or sex independent of DS. Future studies should include demographic variables in their feature space or perform stratified analyses to identify DS-specific metabolic signatures.

## 5 Conclusion

ML and XAI applied to the present metabolomics study identified significant metabolic differences between T21 and D21 groups. This research applied SHAP explainable analysis to discover new biomarkers linking oxidative stress and mitochondrial dysfunction in patients with DS through high-performance tree-based models KTBoost and XGBoost. The levels of amino acids, vitamin C, taurolithocholic acid and thymidine showed significant changes, which may reflect defects in the control systems for oxidative stress, bile acid metabolism and cell activities. Based on metabolomics, DS diagnosis seems possible; the accuracy of one model, KTBoost, in distinguishing T21 from D21 groups is also high. The biomarkers identified by SHAP analysis explain the basis of pathogenesis, were determined to be significant and included L-Citrulline, Kynurenine, Prostaglandin A2/B2/J2, Urate and Pantothenate. However, these results help to clarify the metabolic profile of DS, but more studies are needed to clarify the definitive conclusions. The findings of this study suggest that the powerful combination of metabolomics and AI algorithms may improve diagnostic tools and treatment options for DS. This study identifies new metabolic biomarkers (e.g., L-Citrulline, Kynurenine) and relates them to the pathophysiology of DS, but requires validation in independent and diverse cohorts. Additionally, it remains unclear whether the metabolic changes observed in DS individuals are causal or consequential. Some changes, such as increased markers of oxidative stress, may be a direct result of genetic trisomies, while others may be compensatory responses to metabolic imbalances. Longitudinal studies monitoring metabolite levels over time and experimental interventions targeting these pathways may help to clarify whether these metabolic perturbations contribute to DS pathology or occur as secondary effects. This is beyond the scope of this study and could be investigated in future studies involving clinical experts in DS. However, upcoming studies will focus on multicenter collaborations to externally validate these findings in a diverse population across geography and ethnicity for generalizability. Longitudinal studies will also assess the stability of these biomarkers over time, and integration with multi-omics data (e.g., genomics, proteomics) will enhance mechanistic understanding. Through these efforts, DS can benefit from metabolomics-driven discoveries and transition to clinical translation.

## Data Availability

The dataset will be made available by the corresponding author upon request.
